# Hierarchical Fibers with a Negative Poisson’s Ratio for Tougher Composites

**DOI:** 10.3390/ma6020699

**Published:** 2013-02-22

**Authors:** Yongtao Sun, Nicola Pugno

**Affiliations:** 1 Laboratory of Bio-Inspired Nanomechanics “Giuseppe Maria Pugno”, Department of Structural, Geotechnical and Building Engineering, Politecnico di Torino, Corso Duca degli Abruzzi 24, Torino 10129, Italy; E-Mail: d024536@polito.it; 2 Laboratory of Bio-Inspired and Graphene Nanomechanics, Department of Civil, Environmental and Mechanical Engineering, University of Trento, via Mesiano 77, Trento I-38123, Italy

**Keywords:** negative Poisson’s ratio (NPR), hierarchy, super carbon nanotubes (STs), auxetic, pull-out, toughness

## Abstract

In this paper, a new kind of hierarchical tube with a negative Poisson’s ratio (NPR) is proposed. The first level tube is constructed by rolling up an auxetic hexagonal honeycomb. Then, the second level tube is produced by substituting the arm of the auxetic sheet with the first level tube and rolling it up. The *N*th (N≥1) level tube can be built recursively. Based on the Euler beam theory, the equivalent elastic parameters of the NPR hierarchical tubes under small deformations are derived. Under longitudinal axial tension, instead of shrinking, all levels of the NPR hierarchical tubes expand in the transverse direction. Using these kinds of auxetic tubes as reinforced fibers in composite materials would result in a higher resistance to fiber pullout. Thus, this paper provides a new strategy for the design of fiber reinforced hierarchical bio-inspired composites with a superior pull-out mechanism, strength and toughness. An application with super carbon nanotubes concludes the paper.

## 1. Introduction

In the last few years, due to their special mechanical and electronic properties, hierarchical covalent two-dimensional (2D) and three-dimensional (3D) networks based on one-dimensional (1D) nanostructures have attracted much research attention. One relevant example is carbon nanotube (CNT) networks, in which carbon nanotubes are covalently connected through different nanojunctions, such as X-, Y-, T-shape [1,2,3,4,5,6], even hierarchically [7,8,9,10,11]. Coluci *et al.* [12] proposed self-similar hierarchical super carbon nanotubes (STs) and showed that they are stable and could present metallic or semiconducting behavior. Then, through fractal and fracture mechanics, Pugno [13] evaluated the strength, toughness and stiffness of the STs-reinforced composites and revealed that the optimized number of hierarchical levels is two, similar to the optimization done by Nature in nacre [14] and in other biological or bio-inspired materials [15]. In addition, different numerical methods have been applied to study the mechanical properties of the STs, such as continuum mechanics [16,17], molecular dynamics [18,19] and molecular structure mechanics [20,21]. These numerical simulations generally show that the elastic moduli of the STs were almost independent from the chirality of the ST, slightly affected by its arm tube chirality and determined mainly by the arm tube aspect ratio [22]; also, with the increase of the hierarchical level, the stiffness and modulus of the STs reduced significantly. Through theoretical analysis and finite element calculations, Wang *et al.* [17] indicated that the stiffness reduction was mainly caused by radial shrinking of STs. The deformation of the STs can be greatly decreased if shrinking is suppressed, therefore, they suggested to fill the STs with a matrix material, emphasizing the importance of the STs-reinforced composites, as initially proposed by Pugno [13].

Since normal STs under tension display shrinkage, the concept of negative Poisson’s ratio (NPR) could also be introduced if we appropriately modify the geometrical structures of the super tubes. Due to their special deformation characteristics, the NPR materials have also drawn considerable attentions in the past years. Many kinds of interesting prototypes have been exploited, such as re-entrant honeycombs [23,24], chiral honeycombs [25,26], re-entrant foams [27,28], microporous polymers, particulate composites, fiber reinforced and laminated composites, auxetic yarns and nanocomposites [29], hybrid materials [30,31,32] and networks of 2D and 3D rigid blocks [33,34,35,36,37,38,39,40,41], *etc.* Compared with conventional materials, the NPR materials could have enhanced compressive strength, shear stiffness, indentation resistance and toughness, self-adaptive vibration damping and shock absorption, reduction in thermal stresses, *etc.* For comprehensive reviews, the reader could refer to [24,29,32,42,43,44,45,46,47]. Moreover, the effect of NPR is not only developed in the above mentioned artificial materials, but also exploited by natural layered ceramics to enhance their deformation capability [48].

With respect to the NPR fibers, it is easy to imagine that under tension, instead of shrinking, they will expand in a perpendicular direction to the loading direction and could thus have some interesting properties, when used as fiber-reinforcements in composites. These properties include enhanced toughness, shear stiffness and pull-out resistance, and so on [24,43,47,49]. 

In this paper, combining the peculiar properties that super tubes show and the auxetic characteristics of NPR materials, a new hierarchical structure, hierarchical tubes with a negative Poisson’s ratio, is proposed. Based on the Euler beam theory, the equivalent elastic moduli of the NPR hierarchical tubes under small deformations are calculated. Such auxetic hierarchical fibers are ideal to increase the pull-out resistance and, thus, the toughness of bio-inspired composites.

## 2. Hierarchical Structures with Negative Poisson’s Ratio

### 2.1. Design of Hierarchical NPR Tubes

**Figure 1:**
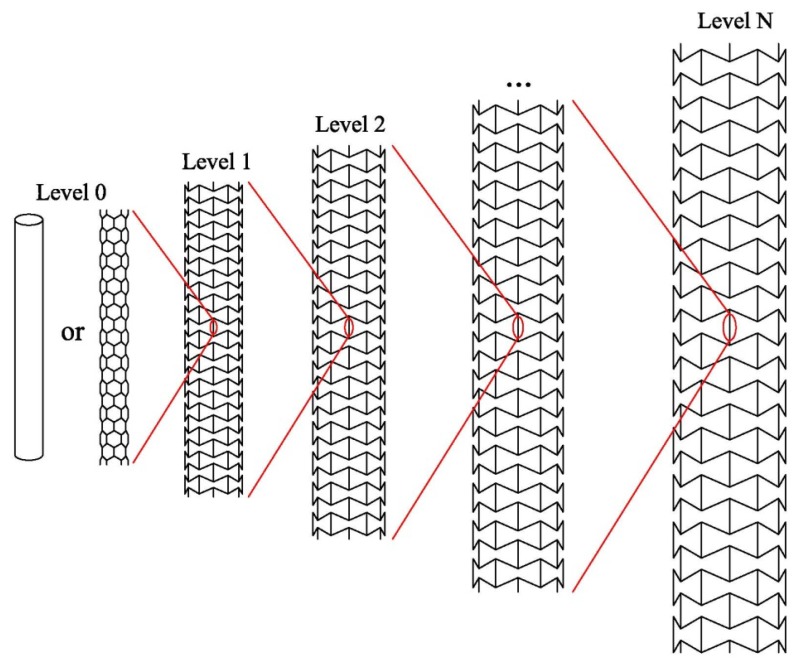
Schematics of the *N*-level hierarchical tube with a negative Poisson’s ratio.

**Figure 2:**
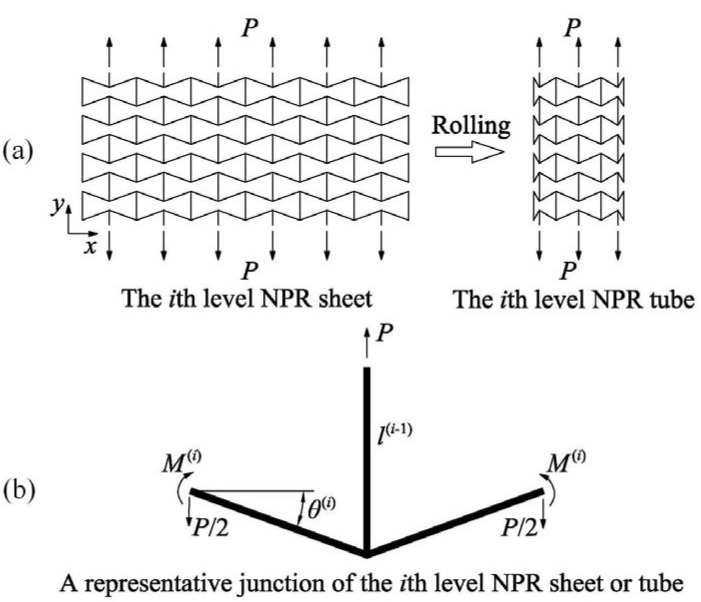
(**a**) Schematics of the *i*th (1≤i≤N) level NPR sheet and the corresponding *i*th (1≤i≤N) level NPR tube made by rolling the NPR sheet; (**b**) the force diagram of a representative junction of the *i*th (1≤i≤N) level NPR sheet or tube subject to the *y*-axis tension.

Figure 1 shows the scheme of a *N*-level (N≥1) hierarchical tube with a negative Poisson’s ratio. The *N*-level ( N≥1 ) hierarchical tube is fabricated through iterating *N* times the process of rolling a NPR sheet to a tube along the *x*-axis, see Figure 1 and Figure 2a.

At first, based on the considered 1D nanostructure (e.g., a solid nanorod or a thin hollow cylinder, such as CNT), the first level NPR sheet that mimics the NPR hexagonal honeycomb is constructed. Then, rolling up the first level NPR sheet gives the first level NPR tube. The second level NPR tube is constructed by substituting the arm tube of the first level NPR sheet with the first level NPR tube and then rolling it up. Iteratively, repeating the above process *N* times, we can build the *N*th level tube. A representative junction of the *i*th (1≤i≤N) level NPR sheet or tube is shown in Figure 2b, in which $l^{\left(i-1\right)}$ is the length of the arms and −30° < θ*^(i)^* < 0°. Similar to the fabrication of hierarchically branched nanotubes [50] and STs [13], that of hierarchical NPR fibers could be realized in the future.

### 2.2. Elasticity of the Hierarchical NPR Tubes

Based on the Euler beam theory, Wang *et al.* [17] derived the equivalent elastic parameters of the STs (positive Poisson’s ratio) from that of the arm tubes and verified the results through finite element simulations. The Young’s modulus E was substituted with the parameter Eβ to describe the equivalent modulus of the CNT and STs, in which β is the thickness to diameter ratio of these thin hollow cylinder tubes. Similarly, in the following, we analytically study the elastic properties of the hierarchical NPR tubes shown in Figure 2a.

#### 2.2.1. The Level 1 NPR Tube

We start by analyzing the level 1 hierarchical NPR sheet and tube under uniaxial tension *p* in the direction *y*, in which the fundamental unit (level 0) is a solid nanorod or a thin hollow cylinder, such as a CNT (Figure 2a). From the force diagram of the representative junction shown in Figure 2b it is evident from the structure periodicity that:

(1) $$M^{\left(1\right)}=\frac{p}{4}l^{\left(0\right)}\cos\theta^{\left(1\right)}$$

in which $M^{\left(1\right)}$ is the bending moment and $l^{\left(0\right)}$ is the arm length.

If the fundamental unit (level 0) is a solid nanorod, we have:

(2) $$\{\begin{array}{c} A^{\left(0\right)}=\frac{1}{4}\pi {\left(d^{\left(0\right)}\right)}^{2}\\ I^{\left(0\right)}=\frac{1}{64}\pi {\left(d^{\left(0\right)}\right)}^{4} \end{array}$$

in which $d^{\left(0\right)}$, $A^{\left(0\right)}$ and $I^{\left(0\right)}$ are its diameter, cross section area and inertia moment. If the fundamental unit (level 0) is a thin hollow cylinder, such as a CNT, denoting its equivalent thickness as $t^{\left(0\right)}$ we have [17]:

(3) $$\{\begin{array}{c} A^{\left(0\right)}=\pi {\left(d^{\left(0\right)}\right)}^{2}\beta^{\left(0\right)}\\ I^{\left(0\right)}=\frac{1}{8}\pi {\left(d^{\left(0\right)}\right)}^{4}\beta^{\left(0\right)} \end{array}$$

in which $d^{\left(0\right)}$, $A^{\left(0\right)}$ and $I^{\left(0\right)}$ are its equivalent diameter, cross section area, inertia moment and $\beta^{\left(0\right)}=t^{\left(0\right)}/d^{\left(0\right)}$ is the thickness-to-diameter ratio.

In order to study the level 1 NPR sheet and tube, we analyze the first level representative junction (Figure 2b). Its lengths along the *x*-axis and *y*-axis are $l_{x}^{\left(1\right)}=2\cos\theta^{\left(1\right)}l^{\left(0\right)}$ and $l_{y}^{\left(1\right)}=\left(1+\sin\theta^{\left(1\right)}\right)l^{\left(0\right)}$. The elongations along the two directions can be obtained through structural analysis; we find:

(4) $$\begin{array}{l} \Delta l_{x}^{\left(1\right)}=\frac{pl^{\left(0\right)}}{E^{\left(0\right)}A^{\left(0\right)}}\sin\theta^{\left(1\right)}\cos\theta^{\left(1\right)}-\frac{p{\left(l^{\left(0\right)}\right)}^{3}}{12E^{\left(0\right)}I^{\left(0\right)}}\sin\theta^{\left(1\right)}\cos\theta^{\left(1\right)}\\ \Delta l_{y}^{\left(1\right)}=\frac{pl^{\left(0\right)}}{E^{\left(0\right)}A^{\left(0\right)}}\left(1+\frac{1}{2}\sin^{2}\theta^{\left(1\right)}\right)+\frac{p{\left(l^{\left(0\right)}\right)}^{3}}{24E^{\left(0\right)}I^{\left(0\right)}}\cos^{2}\theta^{\left(1\right)} \end{array}$$

in which $E^{\left(0\right)}$ is the Young’s modulus of the fundamental unit (level 0). Then, the equivalent strains along the two directions can easily be calculated as:

(5) $$\begin{array}{ll} \varepsilon_{x}^{\left(1\right)} & =\frac{\Delta l_{x}^{\left(1\right)}}{l_{x}^{\left(1\right)}}=\frac{p\sin\theta^{\left(1\right)}}{2E^{\left(0\right)}}\left(\frac{1}{A^{\left(0\right)}}-\frac{{\left(l^{\left(0\right)}\right)}^{2}}{12I^{\left(0\right)}}\right)\\ & =\{\begin{array}{l} \frac{2p\sin\theta^{\left(1\right)}}{3E^{\left(0\right)}\pi {\left(d^{\left(0\right)}\right)}^{2}}\left(3-4{\left(\alpha^{\left(0\right)}\right)}^{2}\right)\;\text{for the solid nanorod}\\ \frac{p\sin\theta^{\left(1\right)}}{6E^{\left(0\right)}\pi {\left(d^{\left(0\right)}\right)}^{2}\beta^{\left(0\right)}}\left(3-2{\left(\alpha^{\left(0\right)}\right)}^{2}\right)\text{for the thin hollow cylinder} \end{array} \end{array}$$

(6) $$\begin{array}{ll} \varepsilon_{y}^{\left(1\right)} & =\frac{\Delta l_{y}^{\left(1\right)}}{l_{y}^{\left(1\right)}}=\frac{p}{E^{\left(0\right)}\left(1+\sin\theta^{\left(1\right)}\right)}\left(\frac{1+1/2\sin^{2}\theta^{\left(1\right)}}{A^{\left(0\right)}}+\frac{{\left(l^{\left(0\right)}\right)}^{2}\cos^{2}\theta^{\left(1\right)}}{24I^{\left(0\right)}}\right)\\ & =\{\begin{array}{l} \frac{4p}{E^{\left(0\right)}\pi {\left(d^{\left(0\right)}\right)}^{2}}\frac{1+1/2\sin^{2}\theta^{\left(1\right)}+2/3{\left(\alpha^{\left(0\right)}\right)}^{2}\cos^{2}\theta^{\left(1\right)}}{1+\sin\theta^{\left(1\right)}}\;\text{for the solid nanorod}\\ \frac{p}{E^{\left(0\right)}\pi {\left(d^{\left(0\right)}\right)}^{2}\beta^{\left(0\right)}}\frac{1+1/2\sin^{2}\theta^{\left(1\right)}+1/3{\left(\alpha^{\left(0\right)}\right)}^{2}\cos^{2}\theta^{\left(1\right)}}{1+\sin\theta^{\left(1\right)}}\text{for the thin hollow cylinder} \end{array} \end{array}$$

in which $\alpha^{\left(0\right)}=l^{\left(0\right)}/d^{\left(0\right)}$ is the aspect ratio of the level 0 arms. Thus, the equivalent Poisson’s ratio $\nu^{\left(1\right)}$ is calculated as:

(7) $$\begin{array}{l} \nu^{\left(1\right)}=-\frac{\varepsilon_{x}^{\left(1\right)}}{\varepsilon_{y}^{\left(1\right)}}\\ =\{\begin{array}{l} \frac{1}{6}\sin\theta^{\left(1\right)}\left(4{\left(\alpha^{\left(0\right)}\right)}^{2}-3\right)\frac{1+\sin\theta^{\left(1\right)}}{1+1/2\sin^{2}\theta^{\left(1\right)}+2/3{\left(\alpha^{\left(0\right)}\right)}^{2}\cos^{2}\theta^{\left(1\right)}}\;\text{for the solid nanorod}\\ \frac{1}{6}\sin\theta^{\left(1\right)}\left(2{\left(\alpha^{\left(0\right)}\right)}^{2}-3\right)\frac{1+\sin\theta^{\left(1\right)}}{1+1/2\sin^{2}\theta^{\left(1\right)}+1/3{\left(\alpha^{\left(0\right)}\right)}^{2}\cos^{2}\theta^{\left(1\right)}}\text{for the thin hollow cylinder} \end{array} \end{array}$$

The level 1 NPR sheet with size $L_{x}^{\left(1\right)}\times L_{y}^{\left(1\right)}$ is constituted by repeating the representative junction (Figure 2b) in its plane $m^{\left(1\right)}$ times along the *x*-axis and $n^{\left(1\right)}$ times along the *y*-axis (Figure 2a). We treat it as a plate with equivalent thickness $t^{\left(1\right)}$, Young’s modulus $E^{\left(1\right)}$ and Poisson’s ratio $\nu^{\left(1\right)}$. By rolling the level 1 NPR sheet in the direction *y* along the longitudinal axis, the level 1 NPR tube is thus obtained. From the equivalence between the circumference of the level 1 NPR tube and the width $L_{x}^{\left(1\right)}$ of the level 1 NPR sheet, it is easy to calculate the equivalent diameter $d^{\left(1\right)}$ of the level 1 NPR tube:

(8) $$d^{\left(1\right)}=L_{x}^{\left(1\right)}/\pi =m^{\left(1\right)}l_{x}^{\left(1\right)}/\pi =2m^{\left(1\right)}l^{\left(0\right)}\cos\theta^{\left(1\right)}/\pi$$

Then, the slenderness ratio $\alpha^{\left(1\right)}$ and the thickness-to-diameter ratio $\beta^{\left(1\right)}$ become:

(9) $$\alpha^{\left(1\right)}=l^{\left(1\right)}/d^{\left(1\right)}=n^{\left(1\right)}l_{y}^{\left(1\right)}/d^{\left(1\right)}=n^{\left(1\right)}\left(1+\sin\theta^{\left(1\right)}\right)\pi /\left(2m^{\left(1\right)}\cos\theta^{\left(1\right)}\right)$$

(10) $$\beta^{\left(1\right)}=t^{\left(1\right)}/d^{\left(1\right)}$$

where $l^{\left(1\right)}$ is the length of the level 1 NPR tube.

Except for the NPR tubes with very small diameters, the slight change of angles between the arms due to rolling can be ignored. Thus, the results obtained for the level 1 NPR sheet are easily extended to the level 1 NPR tube. Accordingly, the total deformation $\Delta L_{y}^{\left(1\right)}$ along the length direction of the level 1 NPR tube can be expressed as:

(11) $$\begin{array}{l} \Delta L_{y}^{\left(1\right)}=n^{\left(1\right)}\Delta l_{y}^{\left(1\right)}=n^{\left(1\right)}\varepsilon_{y}^{\left(1\right)}\left(1+\sin\theta^{\left(1\right)}\right)l^{\left(0\right)}\\ =\{\begin{array}{l} \frac{4n^{\left(1\right)}pl^{\left(0\right)}}{E^{\left(0\right)}\pi {\left(d^{\left(0\right)}\right)}^{2}}\left(1+1/2\sin^{2}\theta^{\left(1\right)}+2/3{\left(\alpha^{\left(0\right)}\right)}^{2}\cos^{2}\theta^{\left(1\right)}\right)\;\text{for the solid nanorod}\\ \frac{n^{\left(1\right)}pl^{\left(0\right)}}{E^{\left(0\right)}\pi {\left(d^{\left(0\right)}\right)}^{2}\beta^{\left(0\right)}}\left(1+1/2\sin^{2}\theta^{\left(1\right)}+1/3{\left(\alpha^{\left(0\right)}\right)}^{2}\cos^{2}\theta^{\left(1\right)}\right)\text{for the thin hollow cylinder} \end{array} \end{array}$$

Thus, the tensional rigidity $k_{y}^{\left(1\right)}$ of the level 1NPR tube is:

(12) $$\begin{array}{l} k_{y}^{\left(1\right)}=\frac{m^{\left(1\right)}p}{\Delta L_{y}^{\left(1\right)}}\\ =\{\begin{array}{l} \frac{m^{\left(1\right)}E^{\left(0\right)}\pi d^{\left(0\right)}}{4n^{\left(1\right)}\alpha^{\left(0\right)}}\frac{1}{1+1/2\sin^{2}\theta^{\left(1\right)}+2/3{\left(\alpha^{\left(0\right)}\right)}^{2}\cos^{2}\theta^{\left(1\right)}}\;\text{for the solid nanorod}\\ \frac{m^{\left(1\right)}E^{\left(0\right)}\pi d^{\left(0\right)}\beta^{\left(0\right)}}{n^{\left(1\right)}\alpha^{\left(0\right)}}\frac{1}{1+1/2\sin^{2}\theta^{\left(1\right)}+1/3{\left(\alpha^{\left(0\right)}\right)}^{2}\cos^{2}\theta^{\left(1\right)}}\text{for the thin hollow cylinder} \end{array} \end{array}$$

Then, the axial rigidity of the level 1 NPR tube can be obtained as:

(13) $$\begin{array}{l} E^{\left(1\right)}A^{\left(1\right)}=k_{y}^{\left(1\right)}l^{\left(1\right)}=k_{y}^{\left(1\right)}n^{\left(1\right)}l^{\left(0\right)}\left(1+\sin\theta^{\left(1\right)}\right)\\ =\{\begin{array}{l} \frac{m^{\left(1\right)}E^{\left(0\right)}\pi {\left(d^{\left(0\right)}\right)}^{2}}{4}\frac{1+\sin\theta^{\left(1\right)}}{1+1/2\sin^{2}\theta^{\left(1\right)}+2/3{\left(\alpha^{\left(0\right)}\right)}^{2}\cos^{2}\theta^{\left(1\right)}}\;\text{for the solid nanorod}\\ m^{\left(1\right)}E^{\left(0\right)}\pi {\left(d^{\left(0\right)}\right)}^{2}\beta^{\left(0\right)}\frac{1+\sin\theta^{\left(1\right)}}{1+1/2\sin^{2}\theta^{\left(1\right)}+1/3{\left(\alpha^{\left(0\right)}\right)}^{2}\cos^{2}\theta^{\left(1\right)}}\text{for the thin hollow cylinder} \end{array} \end{array}$$

That is to say:

(14) $$\frac{E^{\left(1\right)}A^{\left(1\right)}}{E^{\left(0\right)}A^{\left(0\right)}}=\{\begin{array}{l} m^{\left(1\right)}\frac{1+\sin\theta^{\left(1\right)}}{1+1/2\sin^{2}\theta^{\left(1\right)}+2/3{\left(\alpha^{\left(0\right)}\right)}^{2}\cos^{2}\theta^{\left(1\right)}}\;\text{for the solid nanorod}\\ m^{\left(1\right)}\frac{1+\sin\theta^{\left(1\right)}}{1+1/2\sin^{2}\theta^{\left(1\right)}+1/3{\left(\alpha^{\left(0\right)}\right)}^{2}\cos^{2}\theta^{\left(1\right)}}\text{for the thin hollow cylinder} \end{array}$$

in which $A^{\left(1\right)}=\pi {\left(d^{\left(1\right)}\right)}^{2}\beta^{\left(1\right)}$ is the equivalent cross section area of the level 1 NPR tube.

The bending rigidity of the level 1 NPR tube can be expressed as [17]:

(15) $$E^{\left(1\right)}I^{\left(1\right)}=\frac{1}{8}{\left(d^{\left(1\right)}\right)}^{2}E^{\left(1\right)}A^{\left(1\right)}$$

Substituting the expression $A^{\left(1\right)}=\pi {\left(d^{\left(1\right)}\right)}^{2}\beta^{\left(1\right)}$ into Equation (13) gives the equivalent modulus $E^{\left(1\right)}\beta^{\left(1\right)}$ of the level 1 tube. If the fundamental unit (level 0) is the solid nanorod:

(16) $$\frac{E^{\left(1\right)}\beta^{\left(1\right)}}{E^{\left(0\right)}}=\frac{\pi^{2}}{m^{\left(1\right)}{\left(\alpha^{\left(0\right)}\right)}^{2}}\frac{1+\sin\theta^{\left(1\right)}}{1+1/2\sin^{2}\theta^{\left(1\right)}+2/3{\left(\alpha^{\left(0\right)}\right)}^{2}\cos^{2}\theta^{\left(1\right)}}\frac{1}{16\cos^{2}\theta^{\left(1\right)}}$$

Or, if the fundamental unit (level 0) is the thin hollow cylinder, we have:

(17) $$\frac{E^{\left(1\right)}\beta^{\left(1\right)}}{E^{\left(0\right)}\beta^{\left(0\right)}}=\frac{\pi^{2}}{m^{\left(1\right)}{\left(\alpha^{\left(0\right)}\right)}^{2}}\frac{1+\sin\theta^{\left(1\right)}}{1+1/2\sin^{2}\theta^{\left(1\right)}+1/3{\left(\alpha^{\left(0\right)}\right)}^{2}\cos^{2}\theta^{\left(1\right)}}\frac{1}{4\cos^{2}\theta^{\left(1\right)}}$$

#### 2.2.2. The Level *N* NPR Tube

The equivalent elastic parameters of any level *N* (N≥1) NPR tube can be recursively derived by repeating the analysis reported in Section 2.2.1.

About the Poisson’s ratio of the level *N* tube, similar to Equation (7), if the fundamental unit (level 0) is the solid nanorod:

(18) $$\nu^{\left(N\right)}=\{\begin{array}{c} \frac{1}{6}\sin\theta^{\left(N\right)}\left(4{\left(\alpha^{\left(N-1\right)}\right)}^{2}-3\right)\frac{1+\sin\theta^{\left(N\right)}}{1+1/2\sin^{2}\theta^{\left(N\right)}+2/3{\left(\alpha^{\left(N-1\right)}\right)}^{2}\cos^{2}\theta^{\left(N\right)}}\;N=1\\ \frac{1}{6}\sin\theta^{\left(N\right)}\left(2{\left(\alpha^{\left(N-1\right)}\right)}^{2}-3\right)\frac{1+\sin\theta^{\left(N\right)}}{1+1/2\sin^{2}\theta^{\left(N\right)}+1/3{\left(\alpha^{\left(N-1\right)}\right)}^{2}\cos^{2}\theta^{\left(N\right)}}\;N\geq 2 \end{array}$$

or if the fundamental unit (level 0) is the thin hollow cylinder:

(19) $$\nu^{\left(N\right)}=\frac{1}{6}\sin\theta^{\left(N\right)}\left(2{\left(\alpha^{\left(N-1\right)}\right)}^{2}-3\right)\frac{1+\sin\theta^{\left(N\right)}}{1+1/2\sin^{2}\theta^{\left(N\right)}+1/3{\left(\alpha^{\left(N-1\right)}\right)}^{2}\cos^{2}\theta^{\left(N\right)}}\;N\geq 1$$

where $-30^{{}^{\circ}}<\theta^{\left(N\right)}<0^{{}^{\circ}}$ and:

(20) $$\alpha^{\left(N-1\right)}=l^{\left(N-1\right)}/d^{\left(N-1\right)}=\{\begin{array}{ll} l^{\left(0\right)}/d^{\left(0\right)} & N=1\\ n^{\left(N-1\right)}\left(1+\sin\theta^{\left(N-1\right)}\right)\pi /\left(2m^{\left(N-1\right)}\cos\theta^{\left(N-1\right)}\right) & N\geq 2 \end{array}$$

is the slenderness ratio of the arms of the *N*th level NPR tube. 

With respect to the axial rigidity $E^{\left(N\right)}A^{\left(N\right)}$ of the level *N* NPR tube, if the fundamental unit (level 0) is the solid nanorod, from Equation (14) it is easy to obtain that:

(21) $$\frac{E^{\left(N\right)}A^{\left(N\right)}}{E^{\left(0\right)}A^{\left(0\right)}}=\{\begin{array}{ll} C_{1} & N=1\\ C_{1}\overset{N}{\underset{i=2}{\Pi }}\left(m^{\left(i\right)}\frac{1+\sin\theta^{\left(i\right)}}{1+1/2\sin^{2}\theta^{\left(i\right)}+1/3{\left(\alpha^{\left(i-1\right)}\right)}^{2}\cos^{2}\theta^{\left(i\right)}}\right) & N\geq 2 \end{array}$$

in which $C_{1}=m^{\left(1\right)}\frac{1+\sin\theta^{\left(1\right)}}{1+1/2\sin^{2}\theta^{\left(1\right)}+2/3{\left(\alpha^{\left(0\right)}\right)}^{2}\cos^{2}\theta^{\left(1\right)}}$ or if the fundamental unit (level 0) is the thin hollow cylinder:

(22) $$\frac{E^{\left(N\right)}A^{\left(N\right)}}{E^{\left(0\right)}A^{\left(0\right)}}=\overset{N}{\underset{i=1}{\Pi }}\left(m^{\left(i\right)}\frac{1+\sin\theta^{\left(i\right)}}{1+1/2\sin^{2}\theta^{\left(i\right)}+1/3{\left(\alpha^{\left(i-1\right)}\right)}^{2}\cos^{2}\theta^{\left(i\right)}}\right)$$

where $-30^{{}^{\circ}}<\theta^{\left(i\right)}<0^{{}^{\circ}}$ and

(23) $$\alpha^{\left(i-1\right)}=l^{\left(i-1\right)}/d^{\left(i-1\right)}=\{\begin{array}{l} l^{\left(0\right)}/d^{\left(0\right)}\;i=1\\ n^{\left(i-1\right)}\left(1+\sin\theta^{\left(i-1\right)}\right)\pi /\left(2m^{\left(i-1\right)}\cos\theta^{\left(i-1\right)}\right)\;i=2,\cdots ,N \end{array}$$

is the slenderness ratio of the arms of the *i*th level NPR tube. 

Similar to Equation (15), the bending rigidity $E^{\left(N\right)}I^{\left(N\right)}$ of the level *N* NPR tube is:

(24) $$E^{\left(N\right)}I^{\left(N\right)}=\frac{1}{8}{\left(d^{\left(N\right)}\right)}^{2}E^{\left(N\right)}A^{\left(N\right)}$$

in which $d^{\left(N\right)}=L_{x}^{\left(N\right)}/\pi =2m^{\left(N\right)}l^{\left(N-1\right)}\cos\theta^{\left(N\right)}/\pi$ is the equivalent diameter of the level *N* NPR tube.

It is also easy to get that, if the fundamental unit (level 0) is the solid nanorod:

(25) $$\frac{E^{\left(N\right)}\beta^{\left(N\right)}}{E^{\left(0\right)}}=\{\begin{array}{ll} C_{2} & N=1\\ C_{2}\overset{N}{\underset{i=2}{\Pi }}\left(\frac{\pi^{2}}{m^{\left(i\right)}{\left(\alpha^{\left(i-1\right)}\right)}^{2}}\frac{1+\sin\theta^{\left(i\right)}}{1+1/2\sin^{2}\theta^{\left(i\right)}+1/3{\left(\alpha^{\left(i-1\right)}\right)}^{2}\cos^{2}\theta^{\left(i\right)}}\frac{1}{4\cos^{2}\theta^{\left(i\right)}}\right) & N\geq 2 \end{array}$$

in which $C_{2}=\frac{\pi^{2}}{m^{\left(1\right)}{\left(\alpha^{\left(0\right)}\right)}^{2}}\frac{1+\sin\theta^{\left(1\right)}}{1+1/2\sin^{2}\theta^{\left(1\right)}+2/3{\left(\alpha^{\left(0\right)}\right)}^{2}\cos^{2}\theta^{\left(1\right)}}\frac{1}{16\cos^{2}\theta^{\left(1\right)}}$ or, if the fundamental unit (level 0) is the thin hollow cylinder:

(26) $$\frac{E^{\left(N\right)}\beta^{\left(N\right)}}{E^{\left(0\right)}\beta^{\left(0\right)}}=\overset{N}{\underset{i=1}{\Pi }}\left(\frac{\pi^{2}}{m^{\left(i\right)}{\left(\alpha^{\left(i-1\right)}\right)}^{2}}\frac{1+\sin\theta^{\left(i\right)}}{1+1/2\sin^{2}\theta^{\left(i\right)}+1/3{\left(\alpha^{\left(i-1\right)}\right)}^{2}\cos^{2}\theta^{\left(i\right)}}\frac{1}{4\cos^{2}\theta^{\left(i\right)}}\right)$$

#### 2.2.3. Effects of the Parameters $\theta^{\left(N\right)}$, $\alpha^{\left(N-1\right)}$ and *N*

**Figure 3:**
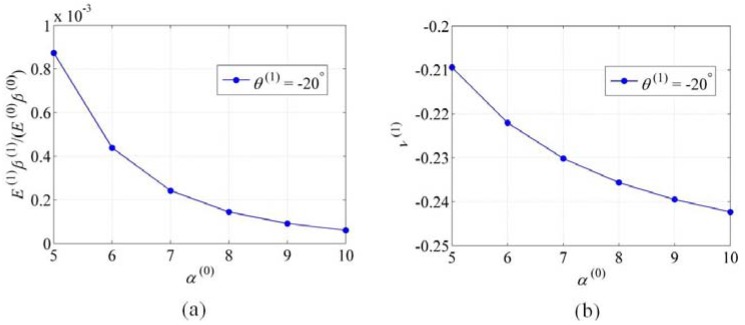
Schematics of (**a**) $E^{\left(1\right)}\beta^{\left(1\right)}$ *vs.* $\alpha^{\left(0\right)}$ and (**b**) $\nu^{\left(1\right)}$ *vs.* $\alpha^{\left(0\right)}$.

**Figure 4:**
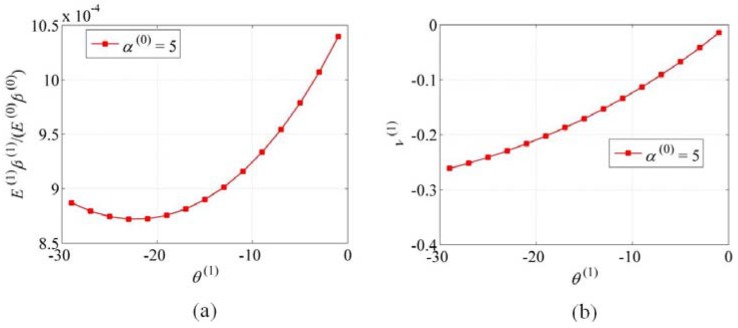
Schematics of (**a**) $E^{\left(1\right)}\beta^{\left(1\right)}$ *vs.* $\theta^{\left(1\right)}$ and (**b**) $\nu^{\left(1\right)}$ *vs.* $\theta^{\left(1\right)}$.

**Figure 5:**
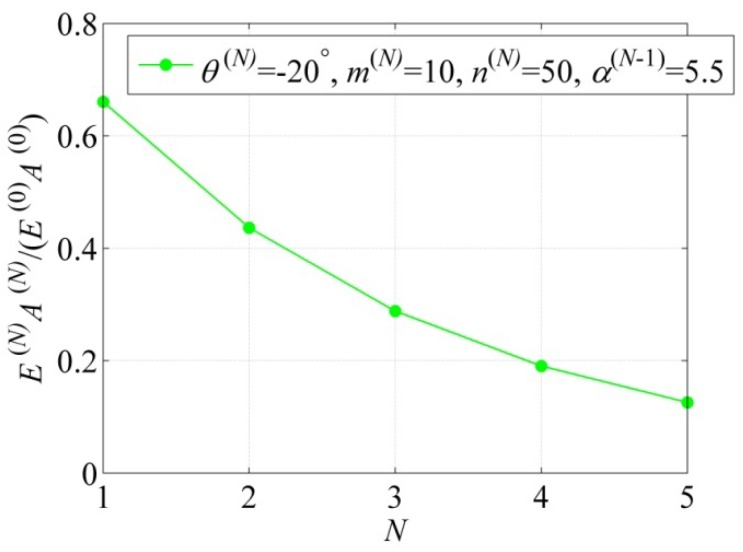
Schematics of the axial rigidity $E^{\left(1\right)}A^{\left(1\right)}$ *vs.* the hierarchical level *N*.

To see the effects of $\theta^{\left(N\right)}$, $\alpha^{\left(N-1\right)}$ and *N* on the elastic properties of the level *N* NPR tube, we use the following examples, considering the NPR tubes composed by a CNT as fundamental unit. With respect to the effects of $\alpha^{\left(N-1\right)}$ on $E^{\left(N\right)}\beta^{\left(N\right)}$ and $\nu^{\left(N\right)}$, the parameters N=1, $\theta^{\left(1\right)}=-20^{{}^{\circ}}$, $m^{\left(1\right)}=10$, $\alpha^{\left(0\right)}=5-10$ are adopted, and the related results are reported in Figure 3. From Figure 3, we can see that both $E^{\left(1\right)}\beta^{\left(1\right)}$ and $\nu^{\left(1\right)}$ decrease with the increase of $\alpha^{\left(0\right)}$. Similarly, for the effects of $\theta^{\left(N\right)}$ on $E^{\left(N\right)}\beta^{\left(N\right)}$ and $\nu^{\left(N\right)}$, the parameters N=1, $\alpha^{\left(0\right)}=5$, $m^{\left(1\right)}=10$, $\theta^{\left(1\right)}=-30^{{}^{\circ}}-0^{{}^{\circ}}$ are considered. The related results are shown in Figure 4. It can be seen that $\nu^{\left(1\right)}$ increases with the increase of $\theta^{\left(1\right)}$, however, $E^{\left(1\right)}\beta^{\left(1\right)}$ at first decreases with the increase of $\theta^{\left(1\right)}$ until about −22°, and after that, it increases with the increase of $\theta^{\left(1\right)}$, showing an interesting minimum that could be invoked for structural optimization; see Figure 4a. For higher level *N* (N>1), the effects of $\theta^{\left(N\right)}$ on $E^{\left(N\right)}\beta^{\left(N\right)}$ and $\nu^{\left(N\right)}$ are similar to that of level 1. Finally, for the effect of the hierarchical level *N* on the axial rigidity $E^{\left(N\right)}A^{\left(N\right)}$, the following parameters are considered: N=1−5, $\theta^{\left(N\right)}=-20^{{}^{\circ}}$, $m^{\left(N\right)}=10$, $n^{\left(N\right)}=50$, $\alpha^{\left(N-1\right)}=5.5$ The related results are displayed in Figure 5. We can see that axial rigidity $E^{\left(N\right)}A^{\left(N\right)}$ decreases with the increase of *N*. Also, for higher level *N* (N>1) NPR tubes, the effects of the parameters $\theta^{\left(N\right)}$, $\alpha^{\left(N-1\right)}$ and *N* are similar to those of the level 1 NPR tube. Note that with the increase of the hierarchical level *N*, the equivalent modulus $E^{\left(N\right)}\beta^{\left(N\right)}$ of the level *N* NPR tube sharply decreases, as can be seen from Equation (25) or (26).

## 3. Conclusions

A new kind of hierarchical tube with a negative Poisson’s ratio is proposed in this paper. The equivalent elastic properties of the NPR hierarchical tubes under small deformations are derived through the Euler beam theory. The results show that both the angles between the arms and the slenderness ratio of the arms have great influences on the equivalent modulus, axial rigidity and Poisson’s ratio of the hierarchical NPR tube and can thus be tuned to match the requirements of a specific application. Under longitudinal axial tension, all levels of the negative Poisson’s ratio hierarchical tubes will expand in the transverse directions rather than shrink. Using these NPR tubes as reinforced fibers in composite materials can result in a higher resistance to fiber pullout and, thus, provides new strategies for the design of bio-inspired fiber reinforced composites with superior toughness. It should be noted that the theory in this paper is limited to the hierarchical NPR tubes with slender arms in small deformations.
